# Successful Arrest of Internal Root Resorption Using Partial Pulpotomy: A Case Report

**DOI:** 10.7759/cureus.71157

**Published:** 2024-10-09

**Authors:** Saeed Asgary

**Affiliations:** 1 Endodontics, Iranian Center for Endodontic Research, Research Institute of Dental Sciences, Shahid Beheshti University of Medical Sciences, Tehran, IRN

**Keywords:** apical periodontitis, calcium-enriched mixture, internal root resorption, irreversible pulpitis, minimally invasive dentistry, pulpotomy, vital pulp therapy

## Abstract

This case report presents a novel, minimally invasive approach for treating internal root resorption (IRR) in a tooth diagnosed with symptomatic irreversible pulpitis and symptomatic apical periodontitis. A 32-year-old patient exhibited rapidly progressing IRR, along with apical radiolucency, one month after the initial diagnosis. Instead of conventional root canal therapy (RCT), a partial pulpotomy was performed using calcium-enriched mixture (CEM) cement with a tampon approach. Over a two-year follow-up period, apical radiolucency healed, with halting of the resorptive area. The root canals gradually narrowed, and no further pathology was detected, resembling the biological outcomes of successful pulpotomies. This case highlights the potential of tampon partial pulpotomy with CEM cement as an effective alternative to conventional RCT, offering improved preservation of pulpal vitality and favorable long-term outcomes.

## Introduction

Irreversible pulpitis and apical periodontitis represent severe inflammatory conditions that compromise tooth vitality and overall oral health. These challenging conditions often necessitate timely intervention to prevent further deterioration of the pulp and periapical tissues [1]. Irreversible pulpitis, characterized by persistent inflammation and symptoms such as spontaneous pain and heightened sensitivity, frequently progresses to apical periodontitis if left untreated. This highlights the critical importance of accurate diagnosis and prompt treatment. Conventional treatment methods, particularly root canal therapy (RCT) using advanced instrumentation methods and minimally invasive techniques, aim to clean/disinfect the root canals and prevent reinfection through three-dimensional filling/sealing the root canal system [2]. However, RCT may sometimes fall short in preserving pulpal vitality and tooth structure, leading to interest in alternative approaches [3].

Internal root resorption (IRR) is a pathological condition of the pulp that results in progressive destruction of the internal dentin walls, leading to the formation of a radiolucent area within the root canal. Clinically, IRR is associated with chronic pulp inflammation and can severely compromise the structural integrity of the tooth. Traditionally, RCT is employed to treat IRR by removing the inflamed pulp and filling the root canal system [4]. However, in cases where the objective is to preserve the vitality of the pulp and maintain the structural integrity of the tooth, minimally invasive approaches such as vital pulp therapy (VPT) may offer better outcomes [5]. Nevertheless, IRR is considered a contraindication for VPT [6].

VPT, which includes procedures such as pulp capping and pulpotomy, has emerged as a conservative alternative to RCT, focused on preserving the vitality of the pulp [7,8]. Among VPTs, pulpotomy in cariously exposed pulp of mature permanent teeth presenting with signs of symptomatic irreversible pulpitis has a high success rate in the long term [9,10]. New approaches, such as the tampon technique, enhance the simplicity and applicability of VPT by achieving hemostasis in cases of excessive bleeding through the application of a biomaterial with mechanical pressure [11]. Recent advances in biomaterials, such as mineral trioxide aggregate and calcium-enriched mixture (CEM) cement, have improved the success rates of VPT by promoting biocompatibility, pulp preservation, and hard tissue formation [12,13]. These biomaterials are critical in enabling minimally invasive treatments aimed at maintaining tooth vitality and functionality, particularly in challenging cases such as IRR.

The aim of this case report is to present a novel, minimally invasive approach for managing IRR in a tooth diagnosed with symptomatic irreversible pulpitis and symptomatic apical periodontitis using partial pulpotomy with CEM cement. By documenting the clinical outcomes, this report highlights the potential of this approach as an effective alternative to conventional RCT, offering enhanced preservation of pulpal vitality and favorable long-term results.

## Case presentation

**Figure 1:**
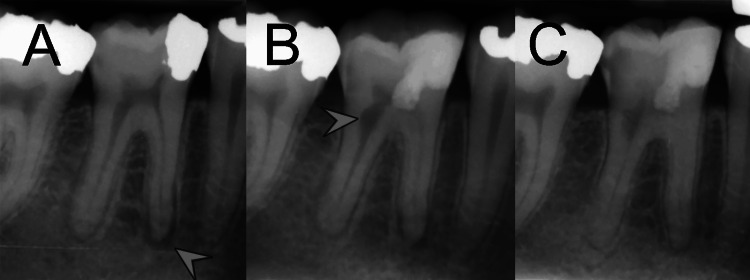
Radiographic progression of the treated case (A) Preoperative periapical radiograph showing a deep amalgam restoration close to the pulp in the lower right first molar, with an apical lesion in the mesial root (arrowhead), PDL widening in the distal root, and large root canals, particularly in the distal root. (B) Immediate postoperative radiograph after partial pulpotomy with CEM cement, revealing an IRR lesion (arrowhead) that developed during the month following the initial diagnosis. (C) Radiograph at 24-month follow-up demonstrates complete apical healing, recalcification of the IRR lesion, narrowing of the root canals, and normal PDL space, with no signs of further pathology. The tooth remained functional and asymptomatic, confirming the successful treatment outcome. PDL, periodontal ligament; CEM, calcium-enriched mixture; IRR, internal root resorption

A 32-year-old female patient presented with complaints of spontaneous pain and sensitivity in the lower right first molar. The symptoms had begun several months after an amalgam restoration and progressively worsened. Clinical examination revealed severe tenderness to percussion, hypersensitivity to vitality tests (especially the cold test), and an amalgam restoration on the mesial aspect of the involved tooth. Radiographic examination showed a deep amalgam restoration likely approaching the pulp, along with an apical lesion on the mesial root, periodontal ligament (PDL) widening in the distal root, and large canals, particularly in the distal root (Figure 1A). The tooth was diagnosed with symptomatic irreversible pulpitis and symptomatic apical periodontitis. The patient's medical history was non-contributory, with no significant systemic conditions.

Treatment options discussed with the patient included tooth extraction, RCT, and VPT. The patient opted for the minimally invasive VPT approach instead of conventional RCT. Due to scheduling constraints, treatment was delayed by one month, during which informed consent was obtained.

At the treatment session, under local anesthesia (lidocaine with epinephrine 1:80,000), after caries removal and pulp exposure, a partial pulpotomy was performed. Using a sterile #4 diamond round bur with copious irrigation, the mesial portion of the coronal pulp was carefully excised. Persistent bleeding was partially controlled by applying normal saline for two minutes, followed by 5.25% sodium hypochlorite for one minute. CEM cement was applied using a tampon approach, which involves the gentle application of a pulp-protecting agent over the amputated pulp tissue to mechanically control the hemorrhage after an unsuccessful attempt to achieve complete hemostasis, similar to its use in medicine [13]. This technique offers a conservative approach to managing pulpitis by creating a protective seal, reducing the need for more invasive procedures such as pulpectomy.

The tooth was then restored with composite resin. Immediate postoperative radiographs revealed that IRR had developed in the month between diagnosis and treatment, as the radiograph showed pathognomonic features of IRR that led us to this diagnosis (Figure 1B).

Follow-up assessments at one day and one-week post-treatment showed that the patient’s symptoms had completely resolved. Regular follow-ups over the subsequent two years included clinical examinations and radiographic assessments. By the 24-month , complete healing of the apical lesion, halting of the resorptive area, and gradual narrowing of the root canals were observed. The PDL appeared normal, and no further pathology was noted (Figure 1C). The tooth remained functional and asymptomatic throughout the follow-up period, indicating the successful management of IRR in a case of symptomatic irreversible pulpitis and symptomatic apical periodontitis.

## Discussion

This case report demonstrates the successful management of IRR in a tooth diagnosed with symptomatic irreversible pulpitis and symptomatic apical periodontitis using a minimally invasive partial pulpotomy technique with CEM cement. The favorable long-term clinical and radiographic outcomes observed over a 24-month follow-up suggest that partial pulpotomy with CEM cement can effectively halt the progression of IRR and facilitate periapical tissue healing, preserving tooth vitality.

The use of CEM cement in this case was pivotal to the success of the treatment. CEM cement is known for its excellent biocompatibility, ability to promote hard tissue formation, and superior sealing properties, which are critical for maintaining a microbe-free environment conducive to pulp healing [14]. Furthermore, the application of CEM cement facilitated the arrest of IRR and the regeneration of calcified tissue, as evidenced by the recalcification observed during follow-up.

This case aligns with a growing body of evidence supporting the use of VPT as an effective alternative to traditional RCT, particularly in cases where preserving pulpal vitality is a priority [15]. Compared to RCT, which often leads to the loss of pulp vitality and the need for extensive restorative procedures, VPT offers a more conservative approach that retains tooth structure and function. Moreover, it is now well established that vital teeth with apical lesions can be successfully treated with VPT, which is an amazing fact today [16].

The tampon approach represents a significant advancement in managing severe pulpitis, especially when rapid hemostasis is difficult to achieve [11]. This technique involves applying pulp-protecting agents such as CEM cement with proper physical pressure over the amputated pulp to control bleeding, fostering an optimal environment for pulp healing. Unlike traditional VPT methods that require full hemostasis, the tampon approach yields comparable outcomes even with less controlled bleeding [3,17,18]. Its simplicity, cost effectiveness, and efficiency make it ideal for busy clinics and pediatric patients [19]. However, further studies are needed to validate its efficacy across diverse populations.

While RCT has long been the standard treatment for cases of irreversible pulpitis and IRR, this case highlights the potential of partial pulpotomy as a viable alternative [20]. The decision to preserve pulp tissue rather than remove it entirely represents a shift toward minimally invasive dentistry, which prioritizes biological principles and tissue preservation. The successful outcome of this case underscores the importance of early diagnosis and intervention in cases of IRR. Timely treatment, combined with the use of advanced biomaterials such as CEM cement, can arrest the progression of resorption and promote healing, resulting in favorable long-term outcomes.

While conventional radiographs were deemed sufficient for diagnosis in this case, the potential utility of cone beam computed tomography imaging in providing enhanced visualization of internal resorption and surrounding structures should not be overlooked, especially in complex cases.

## Conclusions

Tampon partial pulpotomy with CEM cement offers a promising alternative to conventional RCT in managing IRR, particularly in cases of symptomatic irreversible pulpitis/apical periodontitis. This minimally invasive approach preserves pulpal vitality, promotes periradicular tissue healing, and halts the resorptive area while having the potential for long-term success with a reduced risk of complications. However, continued follow-up is warranted to assess the stability of these successful outcomes and to further evaluate the broader applicability of this technique in clinical practice.
